# Etherization

**Published:** 1876-08

**Authors:** 


There is no doubt that but etherization is slowly gaining ground in Europe, and that the day is approaching when chloroformic anæsthesia will be a thing of the past. Meanwhile it is interesting to note the important defections which are continually taking place from the ranks of the chloroformists. Mr. Pallock, of St. George's Hospital, is known to be a most ardent advocate of etherization. Sir James Paget, writing in 1875, says: "For the last two years I have used only sulphuric ether, or, for short operations, nitrous oxide gas or ether spray." Sir Henry Thompson, who used generally to dispense with anæsthesia during lithotrity, now has his patients etherized by Clover's system, which consists in the use of nitrous oxide, followed by ether. "The rapidity and greater safety of the process," he says, "as compared with that by chloroform, together with the freedom from subsequent sickness usually attained—the latter an advantage of no slight value—have influenced my practice, and I now make anæsthesia the rule, and not the exception." Professor Vulpian, of Paris, the eminent experimental physiologist, prefers ether for anæsthesia in vivisection, no doubt from motives of economy, as well as for reasons of a more scientific nature. Speaking of the sudden death of a dog which had been chloroformed for purposes of experiment during a lecture, he says: "This accident is so liable to occur when chloroform is used to produce insensibility in animals about to be experimented upon, that we almost always use sulphuric ether, of which the action is much less dangerous, and less treacherous.—*Boston Med. and Surg. Jour.*

